# Energy-dense diets increase FGF23, lead to phosphorus retention and promote vascular calcifications in rats

**DOI:** 10.1038/srep36881

**Published:** 2016-11-14

**Authors:** Ana I. Raya, Rafael Rios, Carmen Pineda, Maria E. Rodriguez-Ortiz, Elisa Diez, Yolanda Almaden, Juan R. Muñoz-Castañeda, Mariano Rodriguez, Escolastico Aguilera-Tejero, Ignacio Lopez

**Affiliations:** 1 Dept Medicina y Cirugia Animal, Universidad de Cordoba, Cordoba, Spain; 2 Laboratorio de Nefrologia. IIS-Fundacion Jimenez-Diaz, REDinREN, Madrid, Spain; 3 Unidad de Lipidos y Aterosclerosis. IMIBIC/Hospital Universitario Reina Sofia and CIBER Fisiopatologia Obesidad y Nutricion (CIBEROBN), Instituto de Salud Carlos III, Spain; 4 Servicio de Nefrologia, REDinREN, IMIBIC/Hospital Universitario Reina Sofia, Cordoba, Spain

## Abstract

Rats with normal renal function (Experiment 1, n = 12) and uninephrectomized (1/2Nx) rats (Experiment 2, n = 12) were fed diets with normal P (NP) and either normal (NF) or high fat (HF). Rats with intact renal function (Experiment 3, n = 12) were also fed NF or HF diets with high P (HP). Additionally, uremic (5/6Nx) rats (n = 16) were fed HP diets with NF or HF. Feeding the HF diets resulted in significant elevation of plasma FGF23 vs rats fed NF diets: Experiment 1, 593 ± 126 vs 157 ± 28 pg/ml (p < 0.01); Experiment 2, 538 ± 105 vs 250 ± 18 pg/ml (p < 0.05); Experiment 3, 971 ± 118 vs 534 ± 40 pg/ml (p < 0.01). Rats fed HF diets showed P retention and decreased renal klotho (ratio klotho/actin) vs rats fed NF diets: Experiment 1, 0.75 ± 0.06 vs 0.97 ± 0.02 (p < 0.01); Experiment 2, 0.69 ± 0.07 vs 1.12 ± 0.08 (p < 0.01); Experiment 3, 0.57 ± 0.19 vs 1.16 ± 0.15 (p < 0.05). Uremic rats fed HF diet showed more severe vascular calcification (VC) than rats fed NF diet (aortic Ca = 6.3 ± 1.4 vs 1.4 ± 0.1 mg/g tissue, p < 0.001). In conclusion, energy-rich diets increased plasma levels of FGF23, a known risk factor of cardiovascular morbidity and mortality. Even though FGF23 has major phosphaturic actions, feeding HF diets resulted in P retention, likely secondary to decreased renal klotho, and aggravated uremic VC.

Energy-dense foods (fast-foods) are implicated in the etiopathogenesis of dysmetabolic syndrome, a significant and growing health problem both in Western and developing countries^1^,^2^. In addition to their high caloric content, these foods are usually rich in phosphate (P), which is widely used as a food additive^3^,^4^. Moreover, the high fat content of fast-food is likely to increase P digestibility^5^, further aggravating P load. Phosphate overload may cause an increase in fibroblast growth factor 23 (FGF23), which is a known factor of cardiovascular risk^6^. Although there is an increasing awareness of the detrimental effects of the P content of fast-food, the influence of the caloric content of the diet on P balance has not been studied in detail.

Phosphate overload may be tolerated when renal function is intact; however, a decline in renal function, which is associated to high-fat intake and type II diabetes^7^, compromises P handling. In fact, in patients with chronic kidney disease (CKD), elevated serum phosphate plays a major role in the development of vascular calcification (VC)^8^. Phosphate promotes VC through a series of mechanisms, including increased serum CaxP product, which leads to precipitation of calcium salts, and phenotypic transdifferentiation of vascular smooth muscle cells (VSMCs) to osteogenic cells^9^,^10^.

We hypothesized that feeding a diet with high caloric content (high fat diet) would cause an increase in FGF23 in an attempt to control P balance, and would promote VC when renal function is impaired. Thus, the objectives of the present study were: (a) to evaluate changes in FGF23 and P balance induced by high fat diets in rats with normal and reduced renal function; and, (b) to investigate the influence of high fat diets on the development of VC in uremic rats.

## Results

### Studies on P balance

Phosphorus balance was studied in rats with intact and reduced (1/2Nx) renal function fed diets with normal P (NP) and either normal fat (NF) or high fat (HF); and in rats with normal renal function fed high P (HP) diets with NF or HF. In all experiments, rats fed HF diets reduced their food intake when compared when rats fed NF diets. Therefore rats fed HF diets ingested less P than rats fed NF diets. However, the ingested P was more readily absorbed in rats fed HF diets than in rats fed NF diets. This resulted in almost the same net absorption of P and thus in a nearly identical P load in NF vs HF groups: rats with intact renal function fed NP, 44.5 ± 3.2 mg/day vs 44.1 ± 2.4 mg/day; 1/2Nx rats fed NP, 42.1 ± 1.6 mg/day vs 47.3 ± 2.0 mg/day; rats with intact renal function fed HP, 94.5 ± 6.5 mg/day vs 95.9 ± 4.2 mg/day. Urinary P excretion was not different in rats fed HF or NF and NP. In rats fed HP, P excretion by urine was lower (p < 0.05) in rats fed HF diet, 85.0 ± 3.6 mg/day, than in rats fed NF diet, 101.3 ± 4.0 mg/day. In rats with intact renal function fed NP, a non-significant tendency to P retention was detected with HF diet, 14.8 ± 1.8 mg/day vs 12.9 ± 2.2 mg/day in rats fed NF diet. Phosphorus retention in rats on HF diets became significant (p < 0.05) in 1/2Nx rats fed NP, 19.3 ± 1.8 mg/day vs 10.0 ± 2.2 mg/day in rats on NF diet, and in rats fed HP, 10.9 ± 4.4 mg/day vs −6.8 ± 5.2 mg/day in rats on NF diet (Table 1).

**Figure 1:**
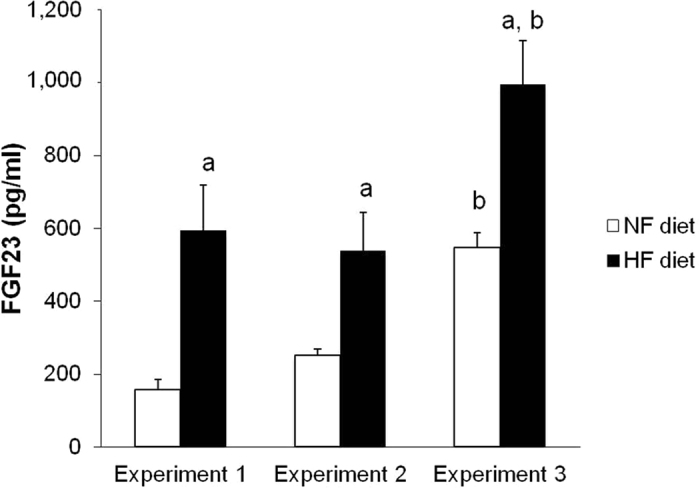
Influence of the fat content of the diet on fibroblast growth factor 23 (FGF23). Plasma concentrations of FGF23 in rats fed normal fat (NF, white bars) or high fat (HF, black bars) diets in the studies on P balance. Experiment 1 (n = 12): rats with intact renal function fed 0.6% P, Experiment 2 (n = 12): 1/2Nx rats fed 0.6% P, Experiment 3 (n = 12): rats with intact renal function fed 1.2% P. ^*a*^p < 0.05 vs NF in the same Experiment, ^*b*^p < 0.05 vs NF in Experiment 1.

In the groups receiving NP, even though P load was similar, rats fed HF diets showed consistently higher plasma FGF23 concentrations than rats fed NF diets: rats with intact renal function fed NP, 593 ± 126 pg/ml vs 157 ± 28 pg/ml (p < 0.01), and 1/2Nx rats fed NP, 538 ± 105 pg/ml vs 250 ± 18 pg/ml (p < 0.05). Feeding a HP diet resulted in higher FGF23 levels than feeding NP diet and rats fed HP-HF diet also had significantly (p < 0.01) elevated FGF23, 995 ± 120 pg/ml, when compared with rats fed HP-NF diet, 547 ± 40 pg/ml (Fig. 1). HF diets elicited increased expression of FGF23 in bone. In a subset of rats fed NP, bone mRNA FGF23/GAPDH ratio increased 3 times in rats fed HF (n = 6) vs rats fed NF (n = 6) diets (3.6 ± 0.9 vs 1.1 ± 0.5, p < 0.05). It is interesting to note that even though plasma FGF23 concentrations were significantly higher in rats fed HF diets than in rats fed NF diets, urinary excretion of P was not increased in the animals fed HF diets (Table 1).

No significant differences between NF and HF groups were found in plasma concentrations of P, iCa, creatinine, total cholesterol, triglycerides and PTH. As expected, 1/2 Nx rats had higher plasma creatinine and urea. Similarly, rats fed HP tended to show higher PTH values. Plasma concentrations of calcidiol were similar in all groups and were not influenced by the increased caloric content in HF diets, except in rats fed HP-HF diet which had higher calcidiol. However, feeding HF diets resulted in significant reductions in plasma concentrations of calcitriol when compared with rats fed NF diets: rats with intact renal function fed NP, 11.3 ± 1.1 pg/ml vs 82.2 ± 11.7 pg/ml (p < 0.001); 1/2Nx rats fed NP, 20.8 ± 10.8 pg/ml vs 117.1 ± 23.7 pg/ml (p < 0.01); rats with intact renal function fed HP, 74.8 ± 25.9 pg/ml vs 143.0 ± 11.8 pg/ml (p < 0.05) (Table 2).

**Figure 2:**
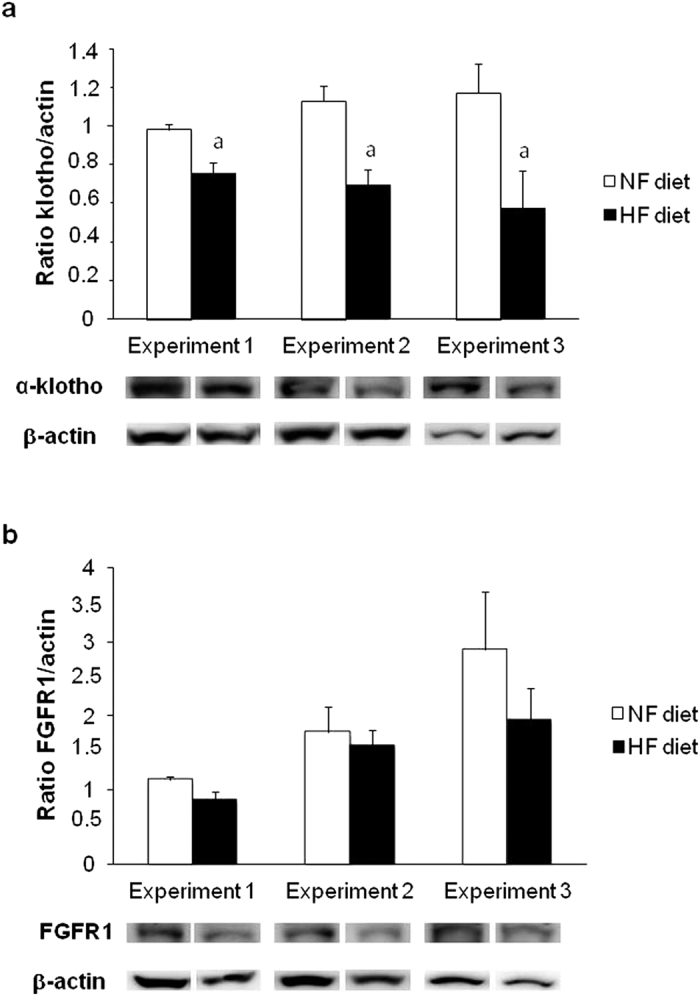
Influence of the fat content of the diet on renal klotho and fibroblast growth factor receptor 1 (FGFR1). (**A**) Western Blots of klotho and (**B**) FGFR1 obtained from renal tissue of rats fed normal fat (NF, white bars) or high fat (HF, black bars) diets in the studies on P balance. Experiment 1 (n = 12): rats with intact renal function fed 0.6% P, Experiment 2 (n = 12): 1/2Nx rats fed 0.6% P, Experiment 3 (n = 12): rats with intact renal funcion fed 1.2% P. ^*a*^p < 0.05 vs NF in the same Experiment.

Figure 2 shows the levels of klotho and FGFR1 proteins in the kidney. Changes in renal klotho were well correlated with changes in plasma FGF23 concentrations. A significant decrease in renal klotho (ratio klotho/β-actin) was identified in all rats fed HF diets: rats with intact renal function fed NP, 0.75 ± 0.06 vs 0.97 ± 0.02 in rats fed NF diet (p < 0.01); 1/2Nx rats fed NP, 0.69 ± 0.07 vs 1.12 ± 0.08 in rats fed NF diet (p < 0.01); and rats with intact renal function fed HP, 0.57 ± 0.19 vs 1.16 ± 0.15 in rats fed NF diet (p < 0.05) (Fig. 2A). Feeding HF diets did not influence FGFR1 expression in the kidney (Fig. 2B).

### Studies on vascular calcification (VC)

**Figure 3:**
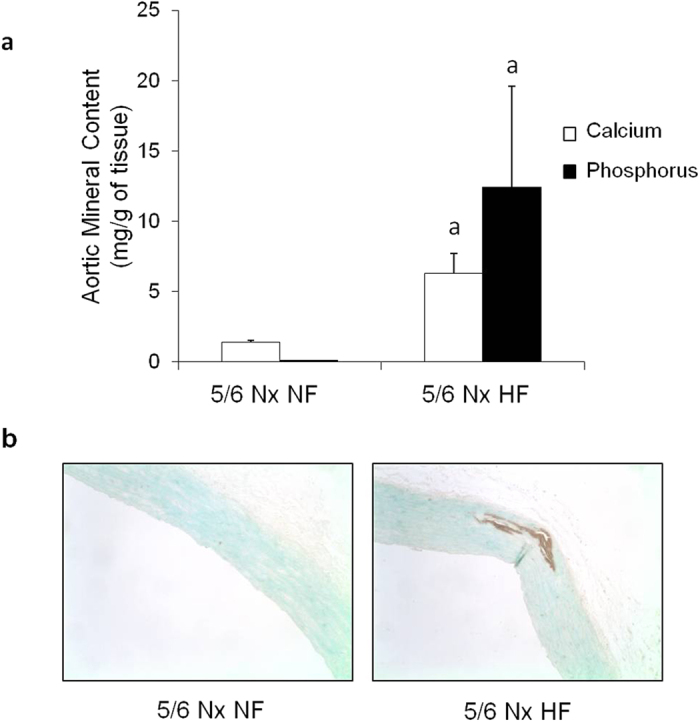
Influence of the fat content of the diet on vascular calcification. (**A**) Calcium (white bars) and phosphorus (black bars) content (mg/g of tissue) in the aortas of uremic (5/6 Nx) rats fed high 1.2% P and either normal fat (NF, n = 6) or high fat (HF, n = 10) diets in Experiment 4. (**B**) Von Kossa stained sections of the aortas of 5/6Nx rats fed high 1.2% P and either NF (left) or HF (right). Calcification foci are stained in brown. ^*a*^p < 0.05 vs NF.

**Figure 4:**
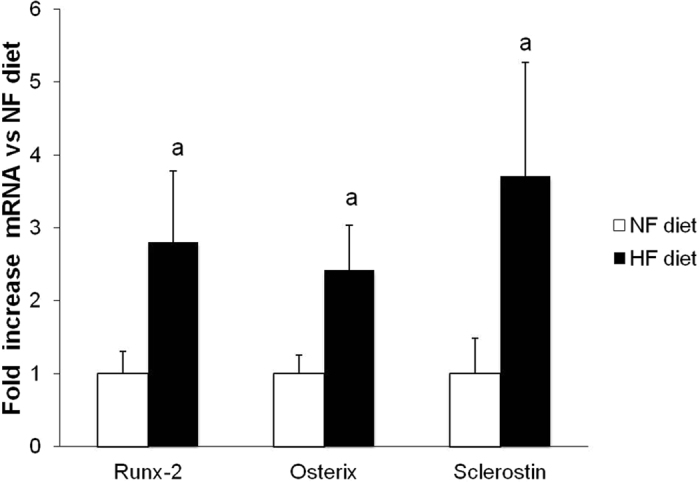
Influence of the fat content of the diet on the expression of osteogenic genes in aortic tissue. Levels of mRNA Runx-2/GAPDH, mRNA Osterix/GAPDH and mRNA Sclerostin/GAPDH in the aortas of uremic (5/6 Nx) rats fed 1.2% P and either normal fat (NF, white bars) or high fat (HF, black bars) diets in Experiment 4 (n = 6 NF and 10 HF). ^*a*^p < 0.05 vs NF.

Uremic rats fed HP-NF diet only experienced very minor mineral deposition in vascular tissue: aortic calcium = 1.4 ± 0.1 mg/g tissue, aortic phosphorus = 0.1 ± 0.1 mg/g tissue. However, 5/6 Nx rats on HP-HF diet showed consistent increases in both aortic calcium (6.3 ± 1.4 mg/g tissue) and phosphorus (12.4 ± 7.2 mg/g tissue), p < 0.001 vs NF diet. Similar results were obtained in other soft tissues and extraskeletal calcification was also confirmed by von Kossa staining (Fig. 3). Feeding HF diets not only resulted in mineral deposition but also in a phenotypic transdifferentiation of the vessel wall that was evidenced by an increase in the expression of osteogenic genes. Thus, mRNA Runx-2/GAPDH ratio was increased by 2.8 fold, mRNA Osterix/GAPDH was increased by 2.4 fold and mRNA Sclerostin/GAPDH was increased by 3.8 fold in rats fed HF diet when compared with rats fed NF diet (Fig. 4).

No differences between uremic rats fed NF and HF diets were observed in plasma creatinine, urea, iCa, P and PTH. Plasma triglycerides, LDL cholesterol and HDL cholesterol tended to be elevated in rats fed HF diet but significant differences with rats fed NF diet were only found in total cholesterol (111.9 ± 6.4 vs 84.2 ± 3.5 mg/dl). Plasma FGF23 was significantly higher (11,366 ± 1,639 vs 4,799 ± 2,402 pg/ml, p < 0.05) in rats fed HF diet (Table 3) and was correlated with aortic calcium (r = 0.640, p = 0.001). The increase in FGF23 was accompanied by a marked but non-significant decrease in the expression of klotho (ratio klotho/β-actin) in the remnant kidney of 5/6 Nx rats, from 1.00 ± 0.39 in rats fed NF diet to 0.55 ± 0.16 in rats fed HF diet.

## Discussion

This study was designed to investigate the effect of feeding energy-dense diets on P balance and VC in rats with normal and reduced renal function. Our results show that rats fed diets with high fat content decreased renal klotho expression, developed P retention due to impaired renal excretion of P, and increased plasma FGF23 concentrations. Moreover, uremic rats fed HF diets showed more severe VC than their normocaloric-fed counterparts.

The study experimental design was based on comparing the effect of diets with identical P concentration and different caloric content. For this purpose a high-fat diet, which is commercialized to induce obesity in rodents, was used. As previously reported^11^, during the experiments the rats fed HF diets reduced food consumption to maintain a constant caloric intake (around 50 kcal/day). Thus, after eating HF or NF diets for 30 days, no differences in body weight were observed between groups. This allowed to study the effect of the caloric content of the diet independent of obesity-mediated mechanisms (e.g. adipokines and other inflammatory mediators released by fat tissue). Although the reduction in food intake in rats fed HF diets resulted in a decreased P intake, in agreement with previous reports^12^ the intestinal absorption of P was increased in rats fed HF diets. The decrease in P intake was compensated by the increase in intestinal absorption of P in rats fed HF diets resulting in almost identical P load in HF vs NF groups.

Feeding HF diets was consistently associated to increases in plasma FGF23 concentrations in all experimental groups. Although FGF23 has been reported to be increased in obese people^13^ and a recent study identified energy intake as a potential predictor of plasma FGF23 concentrations^14^, to the best of our knowledge this is the first report demonstrating a direct relationship between ingestion of a high calories/high fat diet and increases in plasma concentrations of FGF23. Circulating levels of FGF23 are a prognostic factor for cardiovascular disease in patients with CKD^15^ and in the general population^16^. Thus, the demonstration of elevated FGF23 after feeding energy dense diets provides additional insight into the deleterious effect of these diets on cardiovascular health.

One of the most intriguing findings of the present work is that the increase in FGF23 elicited by HF diets was not accompanied by an increase in renal excretion of P. These data strongly suggest resistance to the phosphaturic action of FGF23. To promote phosphaturia in the kidney tubule, FGF23 needs to bind to the klotho-FGFR complex^17^. Therefore, the demonstration of reduced klotho protein levels in the kidneys of rats fed HF diet may explain the resistance to the phosphaturic action of FGF23 found in the present study. Renal klotho has been reported to be decreased in hyperlipidemic ApoE knockout mice and the authors suggested that hyperlipidemia-associated kidney injury might be responsible for the decreased renal expression of klotho^18^. While the renal toxicity of high fat diets is well documented^19^, additional mechanisms might be involved in the decrease of klotho induced by feeding HF diets. Klotho intervenes in energy regulation at different levels and klotho knockout mice display a lean phenotype^20^. Moreover, rats subjected to caloric restriction have been shown to increase renal klotho mRNA expression and klotho protein^21^. Thus, in addition to renal toxic effects, energy-dense diets may directly downregulate renal klotho. Klotho down-regulation by high caloric intake could have significant health consequences since, as shown in this study, it will lead to impaired renal excretion of P and P retention.

Feeding HF diets resulted in low plasma calcitriol concentrations and calcitriol was significantly lower in rats fed HF diets than in rats fed NF diets. The vitamin D content was identical in HF and NF diets. As explained above, the rats fed HF diets ingested less food and this might have resulted in decreased vitamin D intake; but, on the other hand, diets with high fat content have been reported to increase vitamin D absorption by the intestine^22^. In any case, plasma calcidiol concentrations were not decreased in rats fed HF diets. Since calcidiol is the vitamin D metabolite that best reflects nutritional intake^23^, the origin of the decreased plasma calcitriol in rats fed HF diets cannot be attributed to decreased dietary intake of vitamin D. The most likely explanation for the decreased calcitriol is a reduction in calcitriol synthesis secondary to the increase in FGF23, which is known to inhibit the 1-alpha-hydroxylase activity in the kidney^24^. The decrease in serum calcitriol after eating diets with high fat content may also have significant health implications because vitamin D deficiency is associated with cardiovascular disease, diabetes and metabolic syndrome^25^,^26^.

Extraskeletal calcification is a common feature in uremic patients and VC represents an important contributor to the high rate of cardiovascular mortality associated to CKD^8^. The inability to eliminate P plays a major role in the development of uremic VC^10^. Data from Experiments 1–3 indicated that HF diets resulted in P retention particularly when rats were fed HP and, to a lesser extent, with moderate (1/2 Nx) reduction in renal function. In Experiment 4 we evaluated the effect of feeding HP-HF diets to uremic (5/6 Nx) rats. In contrast to rats fed HP-NF diets, uremic rats fed HP-HF diets showed upregulation of osteogenic genes in vascular tissue and marked VC. In addition to P retention, feeding HF diets also resulted in moderate dislipidemia which may also have contributed to the development of VC. Our data demonstrate that although P retention associated to the ingestion of fast-food can be tolerated when renal function is normal, in individuals with a significant decrease in renal function ingestion of high-fat diets may lead to VC.

In conclusion, after being fed with high fat diets rats showed decreased renal klotho expression, increased circulating levels of FGF23 and decreased plasma concentrations of calcitriol. Reduced klotho resulted in resistance to the phosphaturic action of FGF23 and P retention. Moreover, uremic rats fed hypercaloric/high fat diets developed more severe extraosseous calcifications than their normocaloric-fed counterparts.

## Methods

### Ethics

All experimental protocols were reviewed and approved by the Ethics Committee for Animal Research of the University of Cordoba (Cordoba, Spain). All protocols were carried out in accordance with the approved guidelines. They followed the guidelines laid down by the Higher Council of Scientific Research of Spain following the normal procedures directing animal welfare (Real Decreto 223/88, BOE of 18 of March) and adhered to the recommendations included in the Guide for Care and Use of Laboratory Animals (US Department of Health and Human Services, NIH) and European laws and regulations on protection of animals, under the advice of specialized personnel.

### Animals, surgical procedures and diets

Three months-old Wistar rats, provided by the Animal Housing Facilities of the University of Cordoba (Cordoba, Spain), were housed with a 12 h/12 h light/dark cycle. Appropriate measures were taken to ensure animal welfare and to address the basic behavioral and physiological needs of rats. Renal function was reduced by either 1/2 nephrectomy (Nx) or 5/6 Nx. The former was performed by unilateral Nx while the 5/6 Nx was accomplished in a two-step procedure that reduces the original renal mass by five-sixths leading to uremia. These procedures are described in detail elsewhere^27^. Briefly, animals were anesthetized using xylazine (5 mg/kg, ip) and ketamine (80 mg/kg, ip). For the first step of the 5/6 Nx, a 5- to 8-mm incision was made on the left mediolateral surface of the abdomen. The left kidney was exposed, and the two poles (2/3 of renal mass) were ablated. The kidney was inspected and returned to an anatomically neutral position within the peritoneal cavity. The abdominal wall and skin incisions were closed with sutures, and the rat was placed back into its home cage. After 1 week of recovery, in the second step, the animal was reanesthetized and a 5- to 8-mm incision was made on the right mediolateral surface of the abdomen. The right kidney was exposed and unencapsulated, the renal pedicle was clamped and ligated, and the kidney was removed. The ligated pedicle was returned to a neutral anatomical position and the abdomen and skin incisions closed with suture materials. The procedure for 1/2 Nx was identical to the second step of the 5/6 Nx. Phentanyl (0.2 mg/kg, ip) was used as analgesic agent.

Diets with two P concentrations were used in the experiments: normal P (0.6%) diet (NP) and high P (1.2%) diet (HP). Independent of their P content, diets had either a normal fat content (NF diet) with a 5% fat concentration that provided Metabolizable Energy = 3518 kcal/kg (Altromin C 1000, AltrominSpezialfutter GmbH, Germany) or a high fat content (HF diet) with a 35% fat concentration that provided Metabolizable Energy = 5241 kcal/kg (Altromin C 1090–60, AltrominSpezialfutter GmbH, Germany). All diets had 0.6% of Ca and 500 IU/g of vitamin D.

## Experimental Design

### Studies on P balance

These experiments were conducted with the rats housed in metabolic cages, allowing daily control of food and water intake and collection of urine and feces. During the first week the animals were adapted to the cages and received the control diet with 0.6% P and normal fat (NP-NF). Then, rats were switched to experimental diets in three Experiments:

Experiment 1- Rats with intact renal function were allotted to 2 groups (n = 6 each). Rats in group 1 were fed normal P and normal fat diet (NP-NF) and rats in group 2 were fed normal P and high fat diet (NP-HF) for 30 days. Water and food intake were recorded daily during the whole experiment. During the last week of the trial feces and urine were collected daily to assess P balance. At day 30, rats were sacrificed by exsanguination under general anesthesia (thiopental, 20 mg/kg, ip) to obtain blood and tissue samples.

Experiment 2- Rats with reduced (1/2 Nx) renal function (n = 12) were fed either normal P and normal fat (NP-NF, n = 6) or normal P and high fat (NP-HF, n = 6) diet for 30 days, following the same protocol as in Experiment 1.

Experiment 3- Rats with intact renal function were fed HP diet (1.2% P) and were allotted to 2 groups (n = 6 each). Rats in group 1 were fed normal fat diet (HP-NF) and rats in group 2 were fed high fat diet (HP-HF) for 30 days, following the same protocol as in Experiments 1 and 2.

### Studies on vascular calcification (VC)

These studies (Experiment 4) were aimed at evaluating the effect of feeding HF diet on the development of VC in uremic rats. Sixteen rats were fed a NP diet with either normal fat content (NP-NF, n = 6) or high fat content (NP-HF, n = 10) for 45 days. At day 45 the first step of the 5/6 Nx was conducted and a week later the 5/6Nx was completed (day 52). After the second step of 5/6 Nx rats were switched to a HP (1.2%) diet. The fat content of the diet was maintained unchanged; thus rats received HP-NF (n = 6) or HP-HF (n = 10) diets. Rats were sacrificed at day 112 to obtain blood and tissue samples.

### Blood chemistries

Blood for chemistry analyses was obtained at the time of sacrifice (intraperitoneal thiopental anesthesia plus exsanguination), from the abdominal aorta. Blood for measurements of ionized calcium levels was collected in heparinized syringes and immediately analyzed using a Ciba-Corning 634 ISE Ca^2+^/pH Analyzer (Ciba-Corning, Essex, England). Afterwards, plasma was separated by centrifugation and stored at −20 °C until assayed. Plasma creatinine, urea, tryglycerides, total cholesterol, HDL cholesterol, LDL cholesterol and phosphorus were measured by spectrophotometry (BioSystems SA, Barcelona, Spain). ELISA tests were used to quantify plasma FGF23 (Kainos Laboratories, Tokyo, Japan) and PTH (Immutopics, San Clemente, CA). Radioimmunoassay (Immunodiagnostic Systems Ltd, Boldon, UK) was used in plasma samples to determine 25-hydroxyvitamin D (calcidiol) and 1,25-dihydroxyvitamin D (calcitriol).

### Urine and feces analysis

Fecal samples were dried, ashed and demineralized with 0.6 mmol/l HNO_3_ solution. Fecal P was measured by inductively coupled plasma mass spectrophotometry (ICP-MS, Perkin Elmer Elan DRC-e, Walthan, Massachusetts, USA). Urinary phosphorus was measured by spectrophotometry (BioSystems SA, Barcelona, Spain). Net intestinal absorption (equation (1)) and P balance (equation (2)) were calculated as follows:


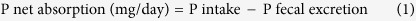



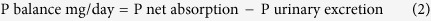


### Protein extraction and Western blot analysis

Proteins were isolated from renal tissue by using a lysis buffer containing HEPES (10 mmol/l), KCl (10 mmol/l), EDTA (0.1 mmol/l), EGTA (0.1 mmol/l), DTT (1 mmol/l), PMSF (0.5 mmol/l), protease inhibitor cocktail (70 μg/ml), and I-Gepal CA-630 (0.6%), pH 7.9 (Sigma Aldrich, St. Louis, MO). Protein concentration was determined by Bradford method. For Western Blot analysis, 50 μg of protein were electrophoresed on a 10% SDS-polyacrilamide gel (Invitrogen, Carlsbad, CA) and electrophoretically transferred (Transfer Systems, BioRad, Hercules, CA) from the gels onto nitrocellulose membranes (Invitrogen). The following steps were perfomed with gentle shaking. Membranes were incubated in TTBS-L solution [20 mMTris-HCl (pH 7.6), 0.2% Tween 20, 150 mMNaCl] (Sigma Aldrich), and 5% nonfat dry milk (Bio-Rad) at room temperature for 1 hour to avoid nonspecific binding. Membranes were then washed with TTBS buffer (the same composition as TTBS-L without nonfat dry milk) and incubated for 2 hours at room temperature with a mouse anti-FGFR1 antibody (GeneTex, Irvine, CA; 0.5 μg/ml) or rat anti-klotho antibody (Alpha Diagnostic Int, San Antonio, TX; 0.5 μg/ml). The membranes were then washed with TTBS buffer and inmunolabeled using a peroxidase-conjugated secondary antibody (1:5000 dilution; Santa Cruz Biotechnology Inc, Santa Cruz, CA). Finally, they were revealed on autoradiographic film using ECL Plus Western Blotting Detection System (GE Healthcare, Piscataway, NJ). Beta-Actin was used as housekeeping protein to ensure equal loading of the gels. Protein levels were quantified using ImageJ software (National Institutes of Health, Bethesda, MD).

### Real-Time-Polymerase Chain Reaction (RT-PCR)

Study of bone FGF23 and aortic Runx-2, Osterix and Sclerostin mRNA was performed by Quantitative Real-Time PCR. Tibial bone or aorta were disrupted using liquid nitrogen and grinded thoroughly with a mortar. Aortic total RNA was extracted from the lysate using RNA extraction kit (RNeasy Fibrous Tissue Mini Kit, Qiagen, Germany). Bone RNA was extracted using the chloroform and isopropanol precipitation method and a treatment with DNAse I Amplification Grade (Sigma-Aldrich). Fifty ng of total RNA were used to analyze mRNA expression in the LightCycler thermal cycler system (Roche Diagnostics, Indianapolis, IN, USA). RT-PCR was performed in one step, using the QuantiTect SYBR Green RT-PCR kit (Qiagen GmbH, Hilden, Germany) following the manufacturer’s protocol. Primers for Runx-2, Osterix and GAPDH were designed with the free Oligo 7 software. The Sclerostin primer was purchased to Integrated DNA Technologies, Inc (San Diego, CA, USA). Primer sequences are listed below:

FGF23- F: 5′-TTGGATCGTATCACTTCAGC-3′,

R: 5′-TGCTTCGGTGACAGGTAG-3′;

Runx-2- F: 5′-CGG GAATGATGAGAACTACTC-3′,

R: 5′-GCG GTCAGAGAACAAACTAGGT-3′;

Osterix- F: 5′-GTACGGCAAGGCTTCGCATCTGA-3′,

R: 5′-TCAAGTGGTCGCTTCGGGTAAAG-3′;

GAPDH- F: 5′-AGGGCTGCCTTCTCTTGTGAC-3′,

R: 5′-TGGGTAGAATCATACTGGAACATGTAG-3′.

The expression of target genes was normalized to GAPDH as housekeeping and calculated according to the 2∆(∆CT) method.

### Assessment of vascular calcification (VC)

Following sacrifice, the thoracic aorta, stomach and lungs were dissected and processed to study mineral content. Calcification was studied by measuring the tissue calcium and phosphorus content. The tissues were demineralized in 10% formic acid (aorta) and 150 mM HCl (stomach and lungs). The calcium and phosphorus content was measured in the supernatant according to a method previously described^27^. Fresh tissue was also fixed in 10% buffered formalin, embedded in paraffin, and cut into 3 μm sections. Paraffin-embedded sections of the aorta, lung and stomach were stained by the Von Kossa method to evaluate mineralization.

### Statistics

Values are expressed as the mean ± standard error (SE). The difference between means for two different groups was determined by t-test; the difference between means for three or more groups was assessed by ANOVA. Fisher LSD test was used as a post-hoc procedure. A correlation study was carried out using the Pearson test. p < 0.05 was considered significant.

## Additional Information

**How to cite this article**: Raya, A. I. *et al*. Energy-dense diets increase FGF23, lead to phosphorus retention and promote vascular calcifications in rats. *Sci. Rep.* **6**, 36881; doi: 10.1038/srep36881 (2016).

**Publisher’s note:** Springer Nature remains neutral with regard to jurisdictional claims in published maps and institutional affiliations.

## Figures and Tables

**Table 1:** Parameters related to phosphorus (P) balance in rats fed diets with normal fat (NF) and high fat (HF) content in Experiments 1–3.

	Experiment 1		Experiment 2		Experiment 3	
	NF (n = 6)	HF (n = 6)	NF (n = 6)	HF (n = 6)	NF (n = 6)	HF (n = 6)
P Intake (mg/day)	93.06 ± 5.15	64.08 ± 3.16^a^	82.60 ± 3.73	66.24 ± 1.48^a^	156.40 ± 7.06	135.00 ± 5.08^a^
P Fecal Excretion (mg/day)	48.59 ± 2.32	20.02 ± 1.41^a^	43.04 ± 1.49	18.09 ± 1.93^a^	61.97 ± 3.42	39.03 ± 1.00^a^
P Net Absorption (mg/day)	44.47 ± 3.23	44.05 ± 2.44	42.14 ± 1.59	47.31 ± 2.04	94.46 ± 6.47	95.93 ± 4.22
P UrinaryExcretion (mg/day)	31.60 ± 1.79	29.25 ± 2.02	29.50 ± 1.82	30.64 ± 1.54	101.29 ± 4.03	85.04 ± 3.63^a^
P Balance (mg/day)	12.86 ± 2.18	14.79 ± 1.81	10.04 ± 2.24	19.34 ± 1.76^a^	−6.83 ± 5.17	10.90 ± 4.44^a^

Experiment 1 (n = 12): rats with intact renal function fed 0.6% P, Experiment 2 (n = 12): 1/2Nx rats fed 0.6% P, Experiment 3 (n = 12): rats with intact renal function fed 1.2% P. ^a^p < 0.05 vs NF in the same Experiment.

**Table 2:** Blood biochemistry in rats fed diets with normal fat (NF) and high fat (HF) content in Experiments 1–3.

	Experiment 1		Experiment 2		Experiment 3	
	NF (n = 6)	HF (n = 6)	NF (n = 6)	HF (n = 6)	NF (n = 6)	HF (n = 6)
Creatinine (mg/dl)	0.58 ± 0.02	0.55 ± 0.02	0.66 ± 0.02^b^	0.72 ± 0.02^b^	0.57 ± 0.01	0.57 ± 0.01
Urea (mg/dl)	22.05 ± 2.75	20.28 ± 1.60	62.85 ± 4.55^b^	51.84 ± 5.97^b^	25.13 ± 2.07	22.94 ± 2.76
Triglycerides (mg/dl)	40.92 ± 5.44	45.11 ± 5.29	39.64 ± 4.61	40.78 ± 3.25	32.17 ± 4.17	40.50 ± 5.66
Total cholesterol (mg/dl)	50.21 ± 3.79	51.57 ± 2.80	48.11 ± 2.69	47.10 ± 2.92	44.71 ± 4.11	52.70 ± 2.92
iCalcium (mmol/l)	1.33 ± 0.01	1.35 ± 0.01	1.28 ± 0.01	1.27 ± 0.01	1.27 ± 0.01	1.26 ± 0.01
Phosphorus (mg/dl)	5.21 ± 0.33	5.93 ± 0.34	5.44 ± 0.21	5.66 ± 0.27	4.98 ± 0.26	6.19 ± 0.52
PTH (pg/ml)	16.99 ± 5.74	11.52 ± 2.88	23.86 ± 6.58	23.44 ± 7.66	25.31 ± 6.10	46.59 ± 14.57^b^
Calcidiol (ng/ml)	30.29 ± 2.33	25.65 ± 3.43	40.85 ± 4.52	42.17 ± 2.21	30.72 ± 2.87	64.34 ± 5.47^a,b^
Calcitriol (pg/ml)	82.19 ± 11.68	11.33 ± 1.11^a^	117.10 ± 23.70	20.78 ± 10.78^a^	143.00 ± 11.78	74.79 ± 25.92^a^

Experiment 1 (n = 12): rats with intact renal function fed 0.6% P, Experiment 2 (n = 12): 1/2Nx rats fed 0.6% P, Experiment 3 (n = 12): rats with intact renal function fed 1.2% P. ^a^p < 0.05 vs NF in the same Experiment, ^b^p < 0.05 vs NF in Experiment 1.

**Table 3:** Blood biochemistry in uremic (5/6 Nx) rats fed 1.2% P and either normal fat (NF) or high fat (HF) diets in Experiment 4.

	Experiment 4	
	NF (n = 6)	HF (n = 10)
Creatinine (mg/dl)	0.91 ± 0.09	1.01 ± 0.10
Urea (mg/dl)	88.56 ± 30.51	80.02 ± 8.72
Triglycerides (mg/dl)	44.71 ± 10.15	58.11 ± 13.88
Total cholesterol (mg/dl)	84.15 ± 3.51	111.88 ± 6.41^a^
LDL cholesterol (mg/dl)	15.68 ± 3.11	18.35 ± 2.06
HDL cholesterol (mg/dl)	37.51 ± 8.39	45.87 ± 3.89
iCalcium (mmol/l)	1.09 ± 0.03	1.11 ± 0.01
Phosphorus (mg/dl)	6.1 ± 1.4	7.8 ± 1.2
FGF23 (pg/ml)	4,799 ± 2,402	11,366 ± 1,639^a^
PTH (pg/ml)	468 ± 116	476 ± 54

^a^p  <  0.05 vs NF.
